# Rural Community Perceptions and Interests in Pharmacogenomics

**DOI:** 10.3390/healthcare8020159

**Published:** 2020-06-05

**Authors:** John Stegelmeier, Christopher Nartker, Charles Barnes, Hugo Rayo, Rebecca Hoover, Julia Boyle, Shanna O’Connor, Jared Barrott

**Affiliations:** College of Pharmacy, Idaho State University, 921 S. 8th Ave., Pocatello, ID 83201, USA; stegjoh2@isu.edu (J.S.); nartchri@isu.edu (C.N.); barnchar@isu.edu (C.B.); rayohugo@isu.edu (H.R.); hoovrebe@isu.edu (R.H.); permjuli@isu.edu (J.B.); oconsha2@isu.edu (S.O.)

**Keywords:** pharmacogenomics, pharmacy, rural communities, survey

## Abstract

Pharmacogenomics testing is a rapidly expanding field with increasing importance to individualized patient care. However, it remains unclear whether the general public in rural areas would be willing to engage in this service. The objective of this survey was to determine rural community-dwelling members’ perceptions of pharmacogenomics. A questionnaire was developed consisting of five Likert-style questions on knowledge and perceptions of pharmacogenomics, a single multiple-choice question on cost of testing, and a free-response question. Two cohorts received the same questionnaire: attendees at a university-sponsored health fair and patients presenting to two independent community pharmacies in southeastern Idaho. While both showed positive reception to the implementation and value of pharmacogenomics, those at the health fair were more in favor of pharmacogenomics, suggesting a need for greater outreach and education to the general public. The findings suggest that interest of rural community-dwelling individuals may be amenable to the expansion of pharmacogenomics testing.

## 1. Introduction

Precision medicine utilizes a patient’s unique genetic makeup, environment, and lifestyle to make healthcare decisions. While still an emerging field, the practice is gaining considerable momentum and study [1]. Pharmacogenomics, where medication use and precision medicine intersect, accounts for the genetic variability of drug response. While multiple factors influence an individual’s response to medications, including age, other medications, organ function, concomitant diseases, and genetics, the latter influences can be predicted to some degree by the use of pharmacogenomics testing [2]. Despite the evidence of gene influence on medication action in the body, the uptake of pharmacogenomics into everyday clinical practice has been slow due to barriers ranging from access in healthcare systems to lack of incorporation into clinical guidelines [3]. A 2019 systematic review on public perceptions of personalized medicine identified that the core themes of study were familiarity, willingness to use, and perceived benefits and risks. The authors found the public to be cautiously optimistic having limited familiarity with precision medicine [4]. These barriers are not unique to pharmacogenomics and the energy to overcome them will need to come from all stakeholders. 

Some of the stakeholders that continue to show hesitation in accepting pharmacogenomics testing are the healthcare providers including physicians and pharmacists. These stakeholders can have a tremendous impact in educating the public about the advantages and disadvantages, but they, too, have shown limited familiarity with the interpretation of pharmacogenomics data and how to counsel a patient regarding results [5,6,7]. Both physicians and pharmacists have expressed positive attitudes towards pharmacogenomics testing, with pharmacists exhibiting greater motivation to learn about it and implement the procedures into clinical practice [8]. However, 40% of pharmacists in another study in 2015 stated they would not use pharmacogenomics testing due to the lack of guidelines on how to implement it [9]. As of July 2016, the Accreditation Council for Pharmacy Education began to require pharmacogenomics as part of the pharmacy education curriculum [10]. Utilizing resources such as the Pharmacogenomics Knowledge Base (PharmGKB) (https://www.pharmgkb.org/) and the Clinical Pharmacogenetics Implementation Consortium (CPIC) (https://cpicpgx.org/guidelines/), which provide guidelines and resources for implementing pharmacogenomics into clinical practice, could help pharmacist with implementation efforts [11,12]. While the technology certainly exists and orders for pharmacogenomics testing are available, the unclear expectations of what a healthcare provider is to do with the information is a consistent theme in survey studies [9,13]. These perceptions are evolving with the breakneck speed of information regarding pharmacogenomics. Studies about the perceptions and barriers five years ago are going to be significantly different than those surveyed in 2020 [14,15]. 

Just as clinical stakeholders have an increased desire to see the implementation of pharmacogenomics testing, so do at-risk patients [16]. Information was a significant determinant in how the lay public perceived pharmacogenomics; however, there were still several concerns identified that made people hesitant to accept pharmacogenomics testing [16,17]. Among the most commonly identified barriers were issues about privacy, discrimination, and being denied medications because of pharmacogenomics testing [16,18]. 

Public reception of pharmacogenomics in rural communities like southeastern Idaho often face unique barriers [19]. The usefulness of pharmacogenomics testing is apparent within large facilities [20] but less study has taken place in rural settings. We know of some of the barriers providers face like unfamiliarity with interpreting and using pharmacogenomics testing [21], yet there is a need to determine public perception in rural communities in order to determine barriers to pharmacogenomics. Some barriers identified by participants of the 1200 Patients Project included the following: lack of understanding of pharmacogenomics, concerns about obtaining insurance coverage and employment, privacy concerns regarding who should have access, and patient/physician relationship [22]. Primary care providers in a rural community (western Montana) thought that patients would be “reluctant and confused” regarding the utilization of pharmacogenomics [19]. Based on our review of current literature, there is a gap in what is known about rural communities’ understanding and perception of pharmacogenomics. We hope that by identifying barriers in the stakeholders of future health professionals and patients, these barriers can then be addressed. In this study, we sought to evaluate both healthcare students’ and patients’ perceptions of pharmacogenomics in a rural community setting to allow for an assessment of a wider variety of rural-dwelling community individuals. 

## 2. Materials and Methods

A 7-item questionnaire was developed for this survey. Idaho State University’s Institutional Review Board deemed the survey exempt from a review (IRB-FY2019-213). The cross-sectional survey consisted of a single open-response question, five 5-point Likert-style questions asking level of agreement on statements regarding understanding of, interest in, applicability of, value of, and helpfulness of pharmacogenomics, and a single multiple-choice question regarding cost. No demographic information or identifiable information was gathered to minimize the time impact of the survey, increase the likelihood of the survey acceptance, and promote participant privacy. To develop the survey, four Doctor of Pharmacy students in their second year each developed and submitted five questions and presented those questions to a pharmacy faculty cohort with expertise in pharmacogenomics and community pharmacy for review and survey adoption (see Table 1). This questionnaire is unique to this study and is not otherwise documented or validated in clinical pharmacy literature. The questions were assessed for utility, appropriateness and readability considering the target population, context, and time commitment of the survey. The questionnaire was evaluated for health literacy concerns using Microsoft Word (Microsoft Office 365, Redmond, WA, USA) via the Flesch–Kincaid Grade Level, scoring on a 10th grade reading level [23]. No incentive was offered for survey participation and completion was voluntary. The questionnaire required a time commitment of approximately 3 min.

### 2.1. Participants

Two cohorts of patients were involved in this study. The first cohort was participants attending a single-day health science-focused research day (symposium) on a university campus. The second cohort were persons presenting to two different community pharmacies located in southeastern Idaho over a period of one month. 

### 2.2. Symposium Cohort

A convenience sample of attendees at the symposium was used for the initial data collection. Participants aged 18 years or older were asked to participate in the survey, there was no exclusion criteria to allow for maximum participation. An informational poster on pharmacogenomics was developed in conjunction with this study as part of the symposium (Figure S1). Participants were approached by study personnel and invited to participate in the study by filling out a questionnaire; personnel also approached attendees at the event who had not yet viewed the poster, offered the questionnaire, and directed participants to the poster. All participants had access to the informational poster either before or during their completion of the questionnaire. Refusals were not recorded. This convenience sample was used for recruitment due to the low cost of the intervention and the anticipated high acceptance rate for participation. 

### 2.3. Community Pharmacy Cohort

Team members offered the questionnaire to all patients who presented for prescription pick-up or drop-off at two local community pharmacies, there was no exclusion criteria to allow for maximum participation. Refusals were not recorded. No additional material (e.g., poster) was presented. The community pharmacy cohort was recruited from local independent pharmacies. The pharmacies were chosen out of convenience to team members and availability of pharmacist mentors. 

### 2.4. Analysis of Data

Likert scale data were analyzed using a non-parametric two-tailed Mann–Whitney U test to compare the mean responses between the symposium cohort and the cohort surveyed at the community pharmacies. GraphPad Prism 7 and Microsoft Excel were used to test the comparisons and graph the data. Agreement was defined as Likert 4 or greater. Survey questions are listed in Table 1. Question 2 required a binary response and data were recalculated into a categorical yes or no response followed by a Fisher’s exact test. Significance was deemed by a *p* value < 0.05. 

## 3. Results

### Survey Results

A total of 85 questionnaires were completed at the university-hosted health science symposium and 52 questionnaires were completed at community pharmacies. A summary of the mean responses for each question is provided in Table 2.

**Figure 1:**
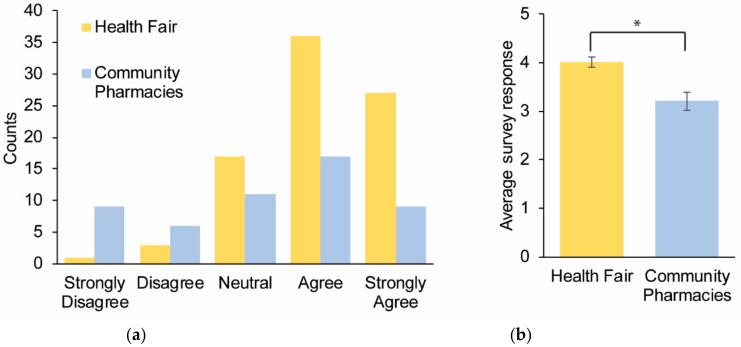
Survey responses to the question: “I understand what pharmacogenomics is”. A total of 137 participants provided responses across multiple sites and were subsequently categorized as the symposium (*n* = 85) and community pharmacy (*n* = 52) participants. (**a**) Cumulative responses for each category depicted by different colors for the two cohorts. (**b**) The average survey response for the two cohorts. Error bars = SEM.

Question 1 was used to determine if participants understood the concept of pharmacogenomics. Seventy-three percent of the symposium and fifty percent of the community pharmacy participants expressed an understanding of pharmacogenomics (Figure 1a). When the two study sites were separated, the survey responses at the symposium demonstrated a statistically higher understanding of pharmacogenomics than the community pharmacy participants: means = 4.0 and 3.2 and medians = 4 and 3.5, respectively (*p* value = 0.001) (Figure 1b).

Question 2 was used to determine if participants were currently taking prescription medications, which might explain any skewing of the value placed on pharmacogenomics. As expected, this question prompted a bimodal response and those surveyed at community pharmacies exhibited a stronger response to the use of prescription medications than those surveyed at the symposium: 85% and 52%, respectively (Fisher’s exact test *p* value = 0.0003). The lack of 100% prescription use among the community pharmacies can be attributed to other family members picking up medications. 

**Figure 2:**
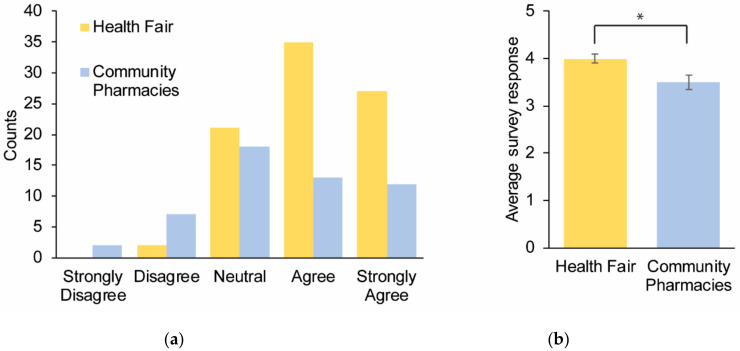
Survey responses to the question: “I am interested in pharmacogenomics”. A total of 137 participants provided responses across multiple sites and were subsequently categorized as the symposium (*n* = 85) and community pharmacy (*n* = 52) participants. (**a**) Cumulative responses for each category depicted by different colors for the two cohorts. (**b**) The average survey response for the two cohorts. Error bars = SEM.

Question 3 was used to determine how interested the participants were in pharmacogenomics. Seventy-two percent of the symposium and 48% of the community pharmacy respondents were interested in pharmacogenomics. There was a statistical difference in the level of interest among health fair participants despite both groups demonstrating high agreement: means = 4.0 and 3.5 and medians = 4 and 3, respectively (*p* value = 0.008) (Figure 2a,b).

**Figure 3:**
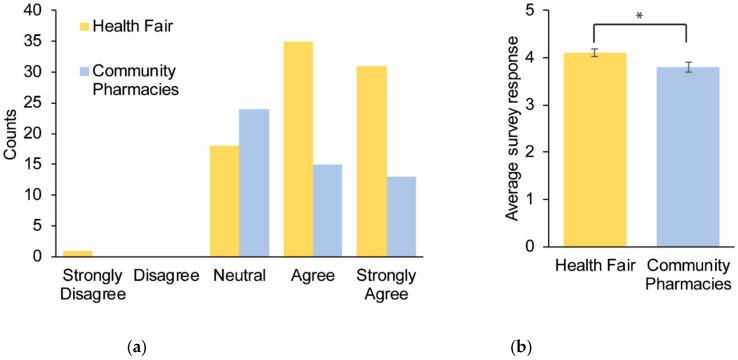
Survey responses to the question: “I believe that pharmacogenomics could help me”. A total of 137 participants provided responses across multiple sites and were subsequently categorized as the symposium (*n* = 85) and community pharmacy (*n* = 52) participants. (**a**) Cumulative responses for each category depicted by different colors for the two cohorts. (**b**) The average survey response for the two cohorts. Error bars = SEM.

Question 4 was used to determine the perceived benefits the participants placed on pharmacogenomics. Seventy-seven percent of the symposium and 28% of the community pharmacy respondents believed that pharmacogenomics could help them. The survey results demonstrated agreement regarding the potential usefulness of pharmacogenomics. The responses from the symposium were more supportive of this statement, but the disparity between the symposium and community pharmacy cohorts was the lowest for all the questions. The average response for the health fair participants was 4.1 (median = 4) and the average response for the community pharmacy participants was 3.8 (median = 4) (*p* value = 0.021) (Figure 3a,b).

**Figure 4:**
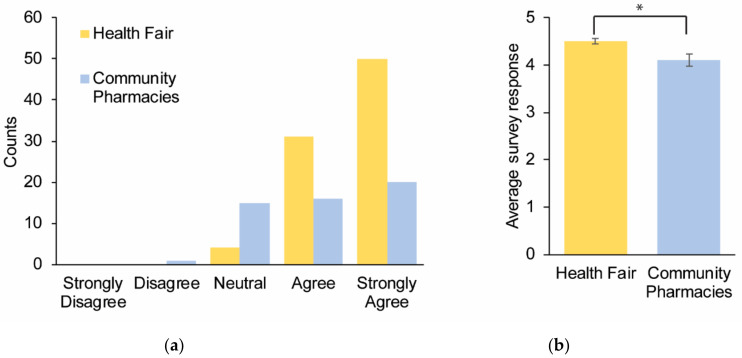
Survey responses to the question: “I believe pharmacogenomics testing is valuable”. A total of 137 participants provided responses across multiple sites and were subsequently categorized as the symposium (*n* = 85) and community pharmacy (*n* = 52) participants. (**a**) Cumulative responses for each category depicted by different colors for the two cohorts. (**b**) The average survey response for the two cohorts. Error bars = SEM.

Question 5 was used to determine the value participants placed on pharmacogenomics testing. Ninety-five percent of the symposium and 69% of the community pharmacy participants believed that pharmacogenomics testing was valuable. There was overwhelming support for this statement with the highest responses being given at 4.5 (median = 5) and 4.1 (median = 4) for the symposium participants and the community pharmacy participants, respectively (*p* value = 0.003). (Figure 4a,b).

**Figure 5:**
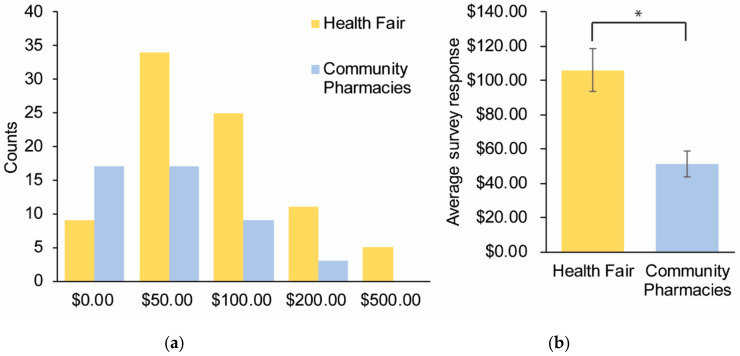
Survey responses to the question: “I would be willing to pay up to $____ out of pocket”. A total of 137 participants provided responses across multiple sites and were subsequently categorized as the symposium (*n* = 85) and community pharmacy (*n* = 52) participants. (**a**) Cumulative responses for each category depicted by different colors for the two cohorts. (**b**) The average survey response for the two cohorts. Error bars = SEM.

Despite the strong agreement that pharmacogenomics testing has value across both study sites, the actual monetary value that participants were willing to pay out of pocket for pharmacogenomics testing appeared to differ depending on the perspective. The symposium participants reported an average of USD 105.95 (median = USD 50.00) value for pharmacogenomics, while the community pharmacy participants were willing to pay USD 51.09 (median = USD 50.00) for pharmacogenomics testing (*p* value = 0.0009) (Figure 5a,b). The inequality in the mean value is most likely because of the five outliers at the health fair that were willing to pay USD 500.00 for pharmacogenomics testing. 

In the open response section of the survey for the symposium participants, there were five comments and 80% were positive and complimentary of pharmacogenomics while one expressed concern about the costs. Among the community pharmacy participants, there were four comments and 75% mentioned unfamiliarity with the concept of pharmacogenomics. 

Lastly, to determine if respondents gave answers that appeared to correlate with one another, we performed linear regression with a Pearson’s coefficient analysis between questions and plotted the R^2^ values in a correlation matrix (Table 3). It is not surprising that questions that evaluated the value (Q5) and helpfulness (Q4) of pharmacogenomics shared the highest correlation (R^2^ = 0.41, *p* value < 0.0001). Additionally, the respondents who see pharmacogenomics as valuable and helpful were also the ones who would pay the most to have that service and information available (R^2^ = 0.09, *p* value = 0.0003). According to the Pearson’s coefficient analysis, any R^2^ value > 0.029 exhibited a *p* value < 0.05.

## 4. Discussion

In the surveyed rural population, generally positive responses in both university-based healthcare-affiliated individuals and community pharmacy patrons were encouraging for the potential expansion of pharmacogenomics into small communities. The patient population is a critical stakeholder and it appears patients have a positive perception of pharmacogenomics in the rural community pharmacy setting.

Increasing the awareness and utility of pharmacogenomics is an important step in further integrating the service into mainstream healthcare. As pharmacogenomics and other genetic testing services becomes more mainstream, it is becoming easier to engage all stakeholders in conversations about the benefits of pharmacogenomics testing. Most studies published on the attitudes of the lay public educated the survey participant prior to giving them the survey [1,3,4,16,18,24,25]. In our rural assessment of patients at community pharmacies, we provided no education on the benefits of pharmacogenomics testing and yet the individuals believed pharmacogenomics could be beneficial to them. There was still a positive perception of how genetic testing could help them in their pharmacotherapy management. While the number of individuals who do not understand what pharmacogenomics is were underrepresented, only three community participants mentioned that they did not know how to define pharmacogenomics. The increased public awareness is likely due to the increasing exposure to direct-to-consumer genetic testing in the public setting. 

The data is still inconclusive on whether genetic testing centers or direct-to-consumer genetic tests such as Ancestry.com (a private genealogy company headquartered in Lehi, UT, USA offering testing to reveal ethnic, historical, and geographic information as well as personalized health reports and family health history) and 23andMe (a private personal genomics and biotechnology company headquartered in Sunnyvale, CA, USA offering DNA testing for ancestry composition/ethnic breakdown as well as traits, and health reports) will drive the transformation in the field of pharmacogenomics testing. While the information provided by direct-to-consumer genetic tests may not be as comprehensive or medically relevant, studies show that consumers place equal value on genetic information that is provided by genetic testing centers and direct-to-consumer tests. [25]. Other researchers argue that genetic testing centers are billable to insurance and will thus provide the most clear pathway to clinical implementation of pharmacogenomics testing [26]. However, the patient empowerment that comes through direct-to-consumer testing provides the most personalized education on genetics [26,27]. 

Limitations of this survey include convenient sampling, lack of recorded declines, lack of demographic information, and likely inherent differences between the university participants and community pharmacy individuals. The community pharmacy-based cohort has the potential to have older individuals who are likely less familiar with the concept of pharmacogenomics compared with the university-based cohort, which likely consisted of younger individuals with potentially more health-centered education on the topic of pharmacogenomics. Attendees at a research symposium may also have heightened interest or exposure to genetic-related sciences than the average community pharmacy participant as those participants were likely students in various health professional programs; this information was not gathered so cannot be confirmed. Health professional students recognize pharmacogenomics as being a useful clinical tool and many report a lack of education to prepare them to utilize it in practice [28,29]; if students did make up a large proportion of the symposium respondents, their results may have been biased based on exposure within their studies. In addition, participants at the symposium were given supplementary educational materials in the form of a research poster, which limits comparability between groups.

The complexity of identifying a drug–gene interaction that has clinical benefit is still being evaluated and continues to cast doubt of finding general patterns of gene variants across a population. However, there is promise such as studies that have analyzed the impact of pharmacogenomics on complex diseases such as depression and anxiety and shown improvements in patient outcomes and a decrease in remission rates in difficult-to-treat mental disorders [30,31]. To move the field of pharmacogenomics forward to treat patients with diseases such as mental disorders, pain, inflammatory disease, cardiovascular disease, or cancer, it is going to require a concerted effort of education. The current barriers to pharmacogenomics are insignificant to the relentless mountain of evidence that continues to grow for the use of pharmacogenomics [32]. As we continue to learn how to use it, it will continue to become more accessible, and costs will continue to drop, thus the barriers will not prevent the research community from assessing the true impact of pharmacogenomics testing. Future research arenas including surveying larger proportions of rural populations are necessary to obtain a more accurate view of public interest in pharmacogenomics. In order to overcome barriers to the role of pharmacogenomics in precision medicine, it would seem that continued patient education of pharmacogenomics may be necessary, so assessments focused on knowledge gaps as well as beliefs may be useful in furthering the science. 

## 5. Conclusions

The findings suggest individuals living in rural communities have a positive perception of pharmacogenomics and may be open to utilizing pharmacogenomics. These positive attitudes are probably influenced by the younger, more educated population and the at-risk, older population that is taking multiple drug prescriptions. Both of these populations were included in our surveys. More studies are needed to evaluate how to best engage patients in utilizing pharmacogenomic tests in their health decision-making. 

## Figures and Tables

**Table 1:** Survey questions regarding pharmacogenomics.

	Strongly Disagree	Disagree	Neutral	Agree	Strongly Agree
(1) I understand what pharmacogenomics is	1	2	3	4	5
(2) I am currently taking a prescription medication	1	2	3	4	5
(3) I am interested in pharmacogenomics	1	2	3	4	5
(4) I believe that pharmacogenomics could help me	1	2	3	4	5
(5) I believe pharmacogenomics testing is valuable	1	2	3	4	5
(6) I would be willing to pay up to $____ out of pocket	$0	$50	$100	$200	$500
(7) Any concerns you may have?					

**Table 2:** A comparison of survey responses between two cohorts using a Mann–Whitney U test.

	Healthcare Students	Pharmacy Patrons	*p* Value
(1) I understand what pharmacogenomics is	4.0 ± 0.11	3.2 ± 0.19	0.001
(2) I am currently taking a prescription medication	52%	85%	0.0003
(3) I am interested in pharmacogenomics	4.0 ± 0.09	3.5 ± 0.15	0.008
(4) I believe that pharmacogenomics could help me	4.1 ± 0.09	3.8 ± 0.11	0.021
(5) I believe pharmacogenomics testing is valuable	4.5 ± 0.07	4.1 ± 0.12	0.003
(6) I would be willing to pay up to $____ out of pocket	$105 ± 12	$51 ± 7	0.0009

**Table 3:** Correlation between survey questions regarding pharmacogenomics.

Q1	Q2	Q3	Q4	Q5	Q6	
1.00	0.01	0.24	0.21	0.16	0.06	Q1
	1.00	0.00	0.01	0.01	0.04	Q2
		1.00	0.31	0.17	0.06	Q3
			1.00	0.41	0.07	Q4
				1.00	0.09	Q5
					1.00	Q6

The shading was added as a quick assessment of relationships.

## Supplementary Materials

The following are available online at https://www.mdpi.com/2227-9032/8/2/159/s1, Figure S1: Poster material presented at the research symposium.
