# Supplementary material for: Rural Community Perceptions and Interests in Pharmacogenomics
Source: Healthcare (Basel). 2020 Jun 5;8(2):159. doi: 10.3390/healthcare8020159 (PMC7348789; doi:10.3390/healthcare8020159)
Supplement: Supplementary file 1 [file healthcare-08-00159-s001.pdf]

Pharmacogenomic Testing in Rural Idaho

John Stegelmeier, Hugo Rayo, Chris Nartker, Charles Barnes, Jared Barrott, Shanna O'Connor, Rebecca Hoover, Julia Boyle
Idaho State University College of Pharmacy

Background: What is Pharmacogenomics?

- Pharmacogenomics: The study of how an individual's genome can impact their responses to medication[1]
- This is an established field of science and an expanding area of clinical practice that is not widely used, particularly in rural areas.
- **Objective:** To highlight pharmacogenomics as a potential service for Idahoans living in rural areas

How is Pharmacogenomics linked to adverse drug reactions?

- Individual patient response to medication can vary as either:
  - ineffective (drug doesn't work)
  - therapeutic (drug works as expected)
  - toxic (drug causes unexpected adverse effects).
- Two individuals can be the same weight and receive same dosage but might vary in drug response.
- Different responses are often linked to genetic differences between patients.

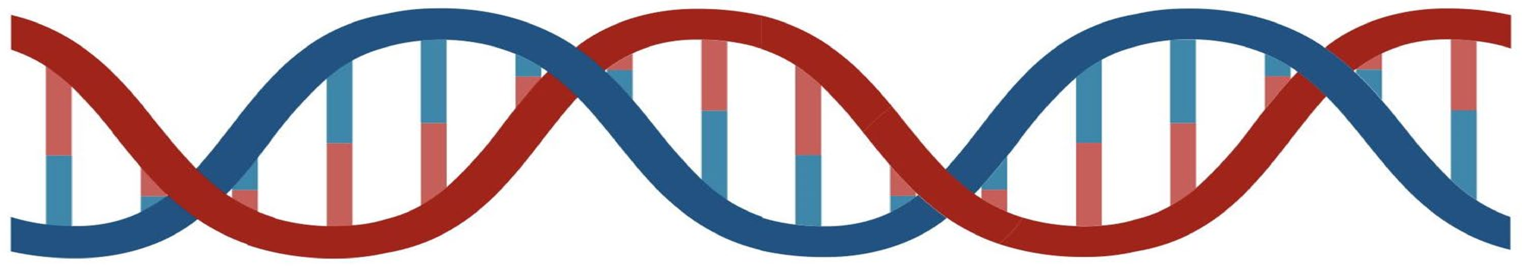

According to the FDA,1 out of 5 injuries or deaths per year to hospitalized patients can be attributed to adverse drug reactions

How effective is the use of Pharmacogenomics in the clinical setting?

- Pharmacogenomic testing has shown benefit in tailoring patients' medications
  - Improved outcomes in psychopharmacology[2]
  - Applications to improve cardiovascular disease treatment
  - Lowering the amount of money wasted in the health industry
- When you have a pharmacogenomic test performed, you are obtaining information about yourself that is already present and may help health care providers make more informed decisions about your treatment.

Discussion and Future Implications

- Pharmacogenomics will likely become the standard of practice in primary and critical care patients and can be carried out in community pharmacies.
- Pharmacists, as drug experts, can help support the health care team by increasing access to pharmacogenomic testing, providing data interpretation, documenting interventions, and working with the patient and other health care team members.
- Limitations:
  - Research about the implementation and cost effectiveness of pharmacogenomics still needs to be done in South East Idaho.
  - Cost of pharmacogenomics and coverage by insurance companies are inconsistent across payers.

Methods: What does the process involve?

1. Patients use an applicator to take a swab of the inside of their cheek to collect cells.
2. Cheek swabs are mailed in or delivered to a lab.
3. The lab uses technology to read the cheek swabs and determine patient's DNA sequence.
4. Lab data is sent to a pharmacist who can interpret the patient's DNA sequence and determine the best medications for the patient.
5. The pharmacist works with the rest of the patient's health care team and the patient to make any needed changes to medications.

Step 1

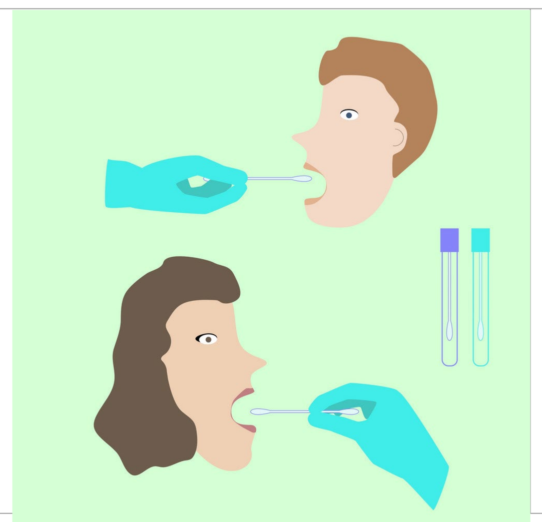

Step 2

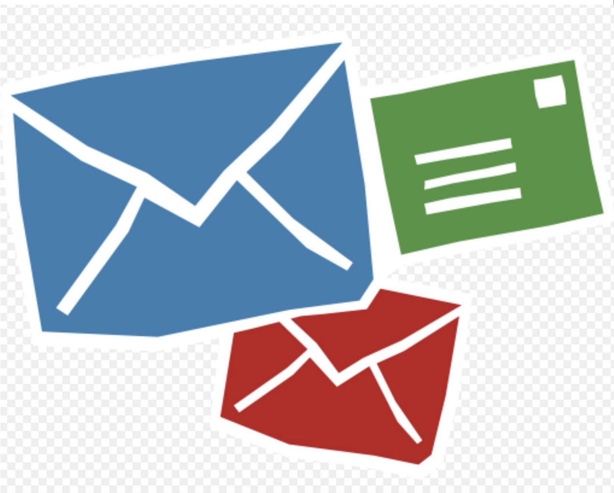

Step 3

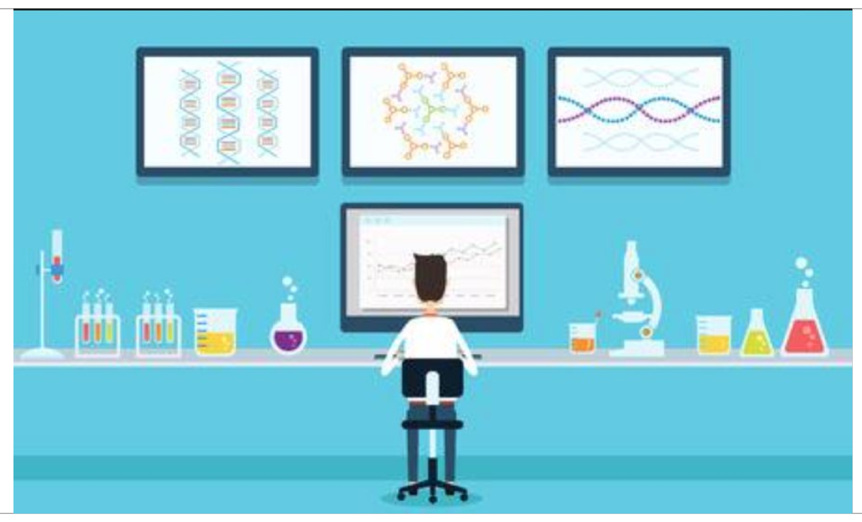

Step 4

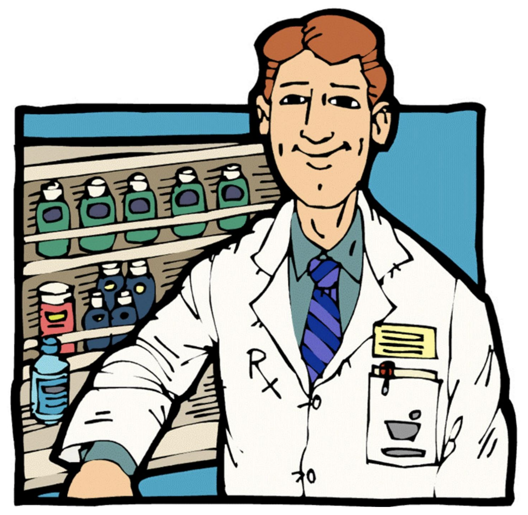

1. Phillips KA, Veenstra DL, Oren E, Lee JK, et al. Potential role of pharmacogenomics in reducing adverse drug reactions: a systematic review. JAMA. 2001 Nov 14;286(18):2270-9.
2. Rigby N, Ma J, Boland J, Van Dorm D, Dowd D, Krause D. 19 Real World Patient-Reported Outcomes Following Pharmacogenomic Testing. CNS Spectr. 2019 Feb;24(1):183.
3. https://www.fda.gov/drugs/developmentapprovalprocess/developmentresources/druginteractionslabeling/ucm110632.htm
